# Specific IgE as the best predictor of the outcome of challenges to baked milk and baked egg

**DOI:** 10.1016/j.jaip.2019.10.039

**Published:** 2020-04

**Authors:** Rachel De Boer, Natalia Cartledge, Sophia Lazenby, Aurelio Tobias, Susan Chan, Adam T. Fox, Alexandra F. Santos

**Affiliations:** a Children's Allergy Service, Guy's and St Thomas' Hospital, London, United Kingdom; b Tommy's National Centre for Miscarriage Research, Institute of Metabolism and Systems Research, College of Medical and Dental Sciences, University of Birmingham, Birmingham, United Kingdom; c Department of Women and Children's Health, School of Life Course Sciences, King's College London, London, United Kingdom; d Peter Gorer Department of Immunobiology, School of Immunology and Microbial Sciences, King's College London, London, United Kingdom; e Asthma UK Centre in Allergic Mechanisms of Asthma, London, United Kingdom

**Clinical Implications**

• Specific IgE performed better than skin prick test to predict reactivity to milk and egg during challenges, with 50% positive predictive value cutoffs being useful to support the decision of whether to challenge to baked milk and egg to assess for tolerance.

Cow's milk and egg allergies are common in childhood, affecting about 1.9% to 3%^1^ and 0.5% to 2.5%^2^ of young children, respectively, but are often outgrown.^3^^,^^4^ Oral food challenge (OFC) is the criterion standard to diagnose milk and egg allergies and to assess resolution; however, OFCs are resource-intensive and involve the risk of inducing an allergic reaction of unpredictable severity. There has been a paradigm shift in the management of milk and egg allergies in view of recent studies showing that about 77% to 83% of milk-allergic patients tolerate baked milk (BM) and 75% of egg-allergic patients tolerate baked egg (BE),^5^^,^^6^ with many centers offering OFCs to assess tolerance to BM and BE as a means of encouraging inclusion of these foods to help broaden the diet, improve quality of life, and possibly assist tolerance acquisition.^6^^,^^7^ In this study, we aimed to define predictors of clinical reactivity during OFCs to milk or egg, both baked and nonbaked, to guide clinical decision making about when to refer for OFC to confirm tolerance.

Clinical records of patients who underwent OFC to cow's milk (baked or fresh) or egg (baked or cooked) between January 2014 and December 2016 were reviewed to assess OFC outcomes compared with skin prick test (SPT) (Stallergenes, Antony, France/ALK-Abelló, Hørsholm, Denmark) and specific IgE (sIgE) (ImmunoCAP, Thermofisher, Uppsala, Sweden) tested before OFC. Referrals for OFCs were made at clinician's discretion, depending on the clinical history and allergy test results. Absence of reaction in the past year, SPT wheal size less than 5 mm and sIgE less than 2 KU/L, or discrepancy between history and allergy test results were the main indications for OFC. OFCs consisted of 4 doses of the challenge food administered openly after a baseline set of observations and physical examination that were repeated about 20 minutes after each dose up to a cumulative amount of 3.6 g of milk protein for BM, 8.33 g of milk protein for fresh milk (FM), 3.7 g of egg protein for BE, and 5.56 g of egg protein for cooked egg (CE) OFCs, as long as no reaction developed. If patients developed signs of an allergic reaction, the OFC was stopped, medication given, and the patients were advised to avoid the food in the diet. See this article's Online Repository at www.jaci-inpractice.org for characteristics of the study population (Table E1) and details about the OFC procedure (Table E2 and Figures E1 and E2) and statistical analyses.

Over the 3-year period, 462 children underwent OFC to milk or egg and 94 (20%) had a positive OFC (Figure 1). Three (2%) of milk and 21 (7%) of egg challenges were inconclusive, due to refusal to eat the entire portion of challenge food and were excluded from the analysis. Overall, the OFCs were well tolerated, with only 6 (1%) requiring intramuscular adrenaline (see Table E3 in this article's Online Repository at www.jaci-inpractice.org): 2 to BM, 1 to FM, and 3 to BE. There was no significant difference in the presence of intermittent or persistent asthma (defined as per Global Initiative for Asthma guidelines) between children who reacted and those who tolerated the food (FM: *P* = .481, BM: *P* = .921, CE: *P* = .476, BE: *P* = .628) or between children who experienced anaphylaxis and those who experienced milder symptoms during OFC.

**Figure 1:**
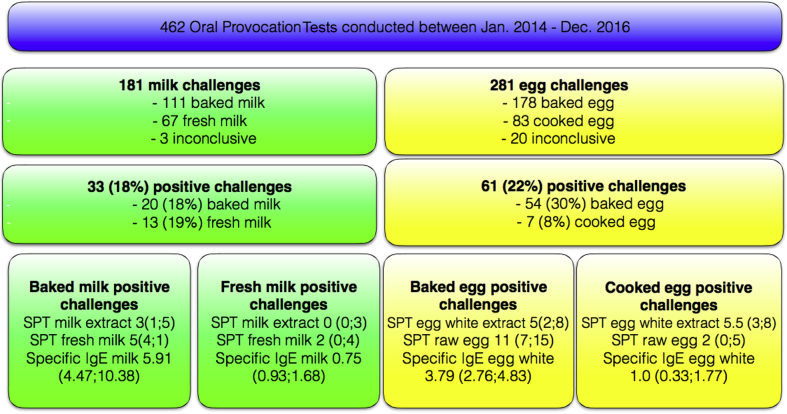
OFCs performed within the 3-year period of the study. For qualitative variables number and percentages and for quantitative variables median and interquartile range are indicated. SPT wheal sizes are represented in mm and specific IgE in KU/L.

The OFC was used as the criterion standard to define allergy to each of the foods tested. We compared the results of SPT and sIgE between subjects who had negative and positive OFC (see Tables E4 and E5 in this article's Online Repository at www.jaci-inpractice.org). Children who reacted to BM had higher SPT wheal size to cow's milk extract and higher milk sIgE. Children who reacted to FM also had higher milk sIgE. In children who reacted to BE, SPT wheal size to egg extract, SPT wheal size to raw egg, and sIgE to egg white were higher compared with those who did not react. Only SPT wheal size to raw egg and the difference in SPT wheal size to egg extract and raw egg were significantly different between CE-allergic and CE-tolerant patients. Data for sIgE to milk and to egg individual allergen components were limited because these are not routinely used in our current clinical practice. Receiver-operator characteristic curve analyses were performed to assess the utility of each test to predict the outcome of OFC and cutoff points with 100% and 50% positive predictive value (PPV) were determined where possible (see Figure E3 and Table E6 in this article's Online Repository at www.jaci-inpractice.org; see also Table I).

**Table I:** Optimal cutoffs for SPT and sIgE to cow's milk or egg white and 100% PPV cutoffs (to confirm allergy) and 50% PPV cutoffs (to determine whether to challenge) for sIgE to cow's milk or egg white∗

Allergy tests	BM allergy	FM allergy	BE allergy	CE allergy
SPT to cow's milk/egg white extract				
AUC ROC 95% CI	0.66 (0.54-0.78)	0.56 (0.37-0.76)	0.6 (0.51-0.68)	0.62 (0.41-0.82)
Optimal cutoffs	2 mm	2 mm	4 mm	3 mm
	S = 76%	S = 29%	S = 57%	S = 43%
	Sp = 55%	Sp = 84%	Sp = 62%	Sp = 81%
	PPV = 27%	PPV = 33%	PPV = 39%	PPV = 20%
	NPV = 91%	NPV = 81%	NPV = 77%	NPV = 93%
SIgE to cow's milk/egg white				
AUC ROC 95% CI	0.72 (0.60-0.84)	0.72 (0.59-0.85)	0.74 (0.64-0.83)	0.75 (0.42-1.00)
Optimal cutoffs	3.06 KU/L	0.3 KU/L	2.81 KU/L	0.94 KU/L
	S = 75%	S = 89%	S = 67%	S = 67%
	Sp = 69%	Sp = 54%	Sp = 81%	Sp = 83%
	PPV = 92%	PPV = 28%	PPV = 84%	PPV = 20%
	NPV = 35%	NPV = 96%	NPV = 61%	NPV = 98%
100% PPV cutoffs	84.9 KU/L (100% PPV)	6.60 KU/L (100% PPV)	ND	ND
50% PPV cutoffs	9.34 KU/L	3.31 KU/L	1.61 KU/L	ND

*AUC ROC*, Area under the receiver-operator characteristic curve; *ND*, not determined; *NPV*, negative predictive value; *S*, sensitivity; *Sp*, specificity.

∗ Optimal cutoffs were defined as the best balance between sensitivity and specificity and calculated on the basis of the largest Youden index.

This was a large study of well-characterized patients, all submitted to OFC, which allowed us to assess the utility of SPT and sIgE to predict the outcome of OFCs and to identify cutoff levels with approximately 50% PPV that can support the decision of when to challenge to BM and BE, in our patient population. This is the first study looking at the clinical utility of SPT and sIgE in the context of BM and BE in our center, which is a World Allergy Organization–accredited allergy center and one of the largest food allergy centers in the world, and one of the very few studies looking at BM and BE OFCs performed in the United Kingdom or Europe. The larger proportion of patients being challenged to BM/BE reflects our recent change in practice of proactively assessing tolerance to the baked forms of these foods as opposed to strict avoidance of all forms of milk and egg advised in the past. In addition, once a BM/BE challenge has been undertaken, it is not common practice in our unit to refer straight on for an FM/CE OFC because the diet has already been expanded and the quality of life improved. Because of the higher number of patients challenged, the cutoffs generated for BM/BE are therefore more robust than for FM/CE. The low rate (18% milk, 22% egg) of positive OFCs may reflect our proficiency in predicting allergic reactivity or alternatively may reflect a more conservative approach to challenge referrals. Our overall anaphylaxis rate was lower than in other series, which is likely to reflect differing criteria for OFC referral and/or practice for administration of adrenaline.

The dosing schedule for BM and BE challenges was based on age-appropriate portions of these foods to ensure a robust definition of patient phenotype and patients' safety when eating shop-bought and home-baked products that can contain variable quantity of the baked allergen. The fact that the proportion of positive OFCs was low despite the higher protein content of the challenge doses further suggests that our population was low risk compared with other published series.^5^^,^^6^^,^^8^^,^^9^

Consistent with previous studies, sex, age, and atopic comorbidities including the presence of asthma were not able to predict clinical reactivity during OFC.^6^^,^^8^^,^^9^ We found that SPT wheal size and sIgE levels were generally higher for children who reacted during OFCs. Surprisingly, SPT did not perform as well as we expected and this could be in part because in our clinic, SPT is at the core of the decision of whether to refer for OFC. Many studies have looked at the ability of allergy tests to predict positive challenges (eg, 95% PPV), which is valuable to confirm allergy, but fewer studies have looked at cutoffs to determine whether to challenge to assess resolution. We identified 50% PPV cutoffs for sIgE milk and egg white in relation to tolerance to BM and BE that can support timely decisions on referral for OFC in the future. Further studies are needed to validate our cutoffs, ideally in a larger prospective study to help establish even more reliable predictors of challenge outcomes.

The study focused on a selected population of children referred for an OFC within our service and does not include all children being assessed for milk and egg allergies; therefore, the cutoff points may have limited generalizability. Compared with other studies,^5^^,^^6^ our cutoffs tended to have lower negative predictive value and higher PPV, suggesting that our population was more likely to react at a given sIgE level compared with other populations. To include a population of children that is more representative of all children with suspected food allergy seen in specialized clinics, we would have had to include highly sensitized patients. However, this would have meant that we had to either challenge children at high risk of reaction, which has ethical limitations, or determine the allergic status of children solely on the basis of SPT and sIgE results and not on the criterion standard OFC, which has its own limitations. This is why our data are important, because they reflect the decision-making process that takes place in a real-life clinic scenario. Only an unbiased approach in a purpose-designed diagnostic study in which patients undergo all tests including OFC would allow for a precise determination of global diagnostic cutoff points for the various tests. The ongoing BAT2 study (NCT03309488) will define more generalizable cutoffs for the diagnosis of BM and BE that allow dispensing OFCs with a higher degree of certainty.

## Figures and Tables

**Figure E1:**
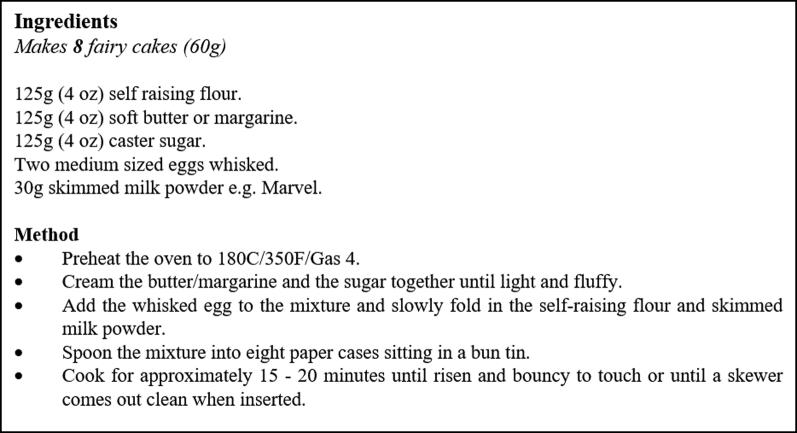
Recipe for standard homemade cupcake for baked milk challenge.

**Figure E2:**
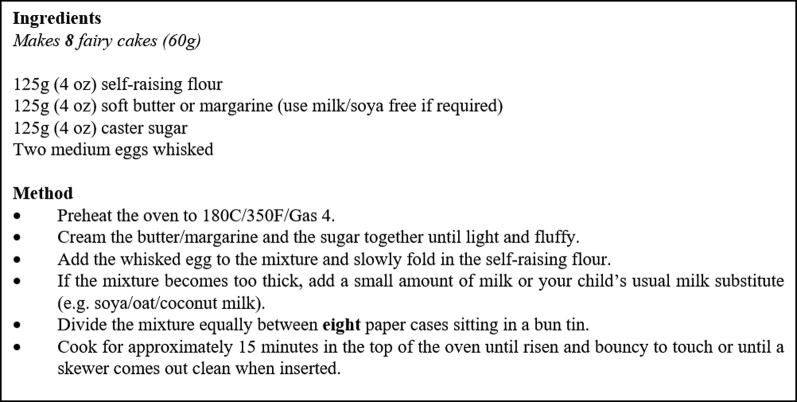
Recipe for standard homemade cupcakes for baked egg challenge.

**Figure E3:**
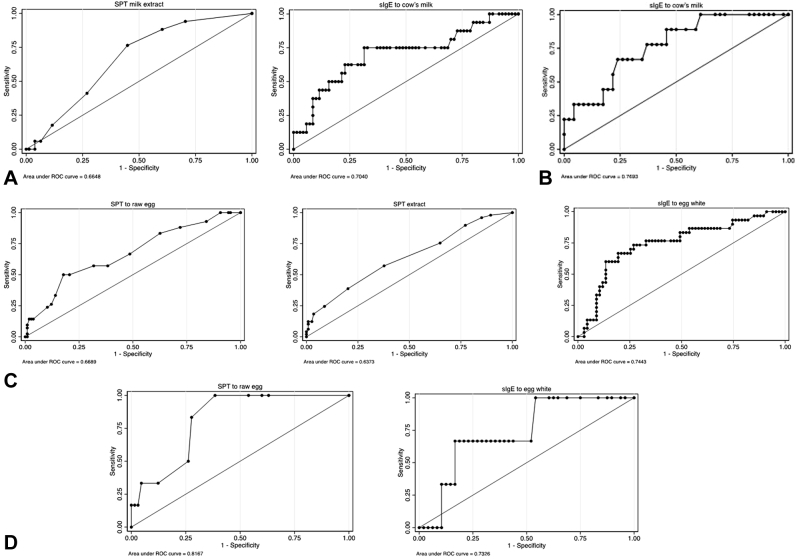
Receiver-operator characteristic (ROC) curves for the different tests regarding (**A**) BM, (**B**) FM, (**C**) BE, and (**D**) CE challenge outcomes. *BE*, Baked egg; *BM*, baked milk; *CE*, cooked egg; *sIgE*, specific IgE.

**Table E1:** Baseline clinical characteristics of the study population assessed for milk or egg allergies

Clinical feature	Milk allergy (n = 181)	Egg allergy (n = 281)
Median age (y)	5.3	6.16
Other food allergy	172 (97)	210 (80)
Atopic eczema	134 (75)	159 (61)
Allergic rhinitis	53 (30)	57 (22)
Persistent asthma	36 (20)	26 (10)
Intermittent asthma	20 (11.2)	51 (19)

Values are n (%) unless otherwise indicated.

**Table E2:** Scheduled doses of milk and egg challenge foods

Challenge food	Doses of challenge food	Dose of food protein (g)
BM∗	Dose 1: ¼ fairy cake	0.33 g milk protein
	Dose 2: ½ fairy cake	0.65 g milk protein
	Dose 3: ¾ fairy cake	0.98 g milk protein
	Dose 4: 1 fairy cake	1.3 g milk protein
	Total cumulative dose: 2½ fairy cakes	3.26 g milk protein
FM	Dose 1: 5 mL	0.17 g milk protein
	Dose 2: 10 mL	0.34 g milk protein
	Dose 3: 30 mL	1.02 g milk protein
	Dose 4: 200 mL	6.8 g milk protein
	Total cumulative dose: 245 mL	8.33 g milk protein
BE∗	Dose 1: ¼ fairy cake	0.47 g egg protein
	Dose 2: ½ fairy cake	0.94 g egg protein
	Dose 3: ¾ fairy cake	1.41 g egg protein
	Dose 4: 1 fairy cake	1.875 g egg protein
	Total cumulative dose: 2½ fairy cakes	3.7 g egg protein
CE	Dose 1: 0.5 g	0.06 g egg protein
	Dose 2: 4 g	0.5 g egg protein
	Dose 3: 10 g	1.25 g egg protein
	Dose 4: 30 g	3.75 g egg protein
	Total cumulative dose: 44.5 g	5.56 g egg protein

*BE*, Baked egg; *BM*, baked milk; *CE*, cooked egg.

∗ For recipes used for BM and BE challenge foods, see Figure E2.

**Table E3:** Characteristics of allergic reactions during OFCs to milk or egg

Symptom	OFC to milk	OFC to egg
Nausea and abdominal pain	6 (18)	28 (46)
Pruritus	12 (36)	27 (44)
Rash	20 (26)	16 (26)
Vomiting	2 (6)	22 (36)
Urticaria	4 (12)	17 (28)
Rhinitis	7 (21)	15 (25)
Possible neurological symptoms∗	5 (15)	7 (11)
Stridor	5 (15)	6 (10%)
Wheeze	6 (18)	1 (2)

Treatment administered	OFC to milk	OFC to egg
Antihistamine	33 (100)	61 (100)
Salbutamol	8 (24)	2 (3)
Intramuscular adrenaline	3 (9)	3 (5)
Prednisolone	1 (3)	2 (3)

∗ Examples of neurological symptoms are dizziness, feeling queasy, and change in behavior.

**Table E4:** Allergy test results comparing children with positive and negative challenges to baked and fresh cow's milk

Patient characteristics	BM challenges (n = 111)			FM challenges (n = 67)		
	Positive, (n = 20)	Negative (n = 91)	*P* value	Positive (n = 13)	Negative (n = 54)	*P* value
Age (y)	4.4 (2.3-8.6)	4.5 (2.3-7.1)	0.860	3.7 (2.6-6.2)	4.3 (2.7-7.3)	.788
History of reactions to milk	35% (7)	40% (36)	0.705	15% (2)	19% (10)	.791
SPT FM (mm)	5 (4-1)	7 (5.5-8.5)	.473	2 (0-4)	2 (1-3)	.907
SPT milk extract (mm)	3 (1-5)	2 (0-4)	**.031**	0 (0-3)	0 (0-1)	.850
SPT difference FM-milk extract (mm)	3 (3-3)	5 (5-5)	.076	0 (0-2)	1 (0-2)	.282
sIgE to milk (KU/L)	5.91 (4.47-10.38)	1.68 (2.10-3.77)	**.011**	0.75 (0.93-1.68)	0.27 (0.22-0.49)	**.011**

*BM*, Baked milk; *sIgE*, specific IgE.

Median, interquartile range, and *P* values using Wilcoxson rank sum test are represented for SPT and sIgE results. *P* values <.05 are marked in bold.

**Table E5:** Allergy test results comparing children with positive and negative challenges to BE and CE

Patient characteristics	BE challenges (n = 179)			CE challenges (n = 83)		
	Positive (n = 54)	Negative (n = 125)	*P* value	Positive (n = 7)	Negative (n = 76)	*P* value
Age (y)	6.0 (3.2-8.2)	4.3 (2.5-8.2)	.157	11.6 (4.9-13.3)	4.1 (2.1-8.4)	.046
History of reactions to egg	11.1% (6)	13.6% (17)	.974	28.57% (2)	28% (21)	.974
SPT raw egg (mm)	11 (7-15)	7 (5-9)	**.001**	5.5 (3-8)	3 (0-6)	**.009**
SPT egg extract (mm)	5 (2-8)	4 (2-6)	**.005**	2 (0-5)	1 (0-3)	.283
SPT difference raw egg-egg extract (mm)	4 (2-6)	3 (2-5)	.862	3.5 (3-4)	0 (0-2)	**.010**
sIgE to egg white (KU/L)	3.79 (2.76-4.83)	1 (0-2.12)	**<.001**	1 (0.33-1.77)	0.31 (0-0.68)	.180

*BE*, Baked egg; *CE*, cooked egg; *sIgE*, specific IgE.

Median and interquartile range are represented for SPT and sIgE results. *P* values <.05 are marked in bold.

**Table E6:** Optimal cutoffs for SPT to FM/raw egg and difference between SPT to FM and SPT to milk/egg extract

Allergy tests	BM allergy	FM allergy	BE allergy	CE allergy
SPT FM/raw egg				
AUC ROC 95% CI	0.56 (0.53-0.6)	0.54 (0.41-0.67)	0.66 (0.5-0.75)	0.81 (0.75-0.87)
	1 mm	4 mm	11 mm	3 mm
	S = 100%	S = 23%	S = 50%	S = 100%
	Sp = 12%	Sp = 85%	Sp = 82%	Sp = 62%
	PPV =19%	PPV = 27%	PPV = 53%	PPV = 19%
Optimal cutoffs	NPV = 100%	NPV = 82%	NPV = 81%	NPV = 100%
Difference between SPT to FM/raw egg and SPT to milk/egg extract				
AUC ROC 95% CI	0.55 (0.46-0.64)	0.5	0.58 (0.49-0.66)	0.77 (0.70-0.84)
	10 mm	12 mm	2 mm	0 mm
	S = 13%	S = 0%	S = 76%	S = 100%
	Sp = 96%	Sp =100%	Sp = 40%	Sp = 54%
	PPV = 40%	PPV = 0%	PPV = 34%	PPV = 20%
Optimal cutoffs	NPV = 85%	NPV = 50%	NPV = 80%	NPV = 100%

*AUC ROC*, Area under the receiver-operator characteristic curve; *BE*, baked egg; *BM*, baked milk; *CE*, cooked egg; *NPV*, negative predictive value; *PPV*, positive predictive value; *S*, sensitivity; *S*p, specificity.

## Online Repository Methods

### Skin prick testing and specific IgE testing

Skin prick test (SPT) was performed as previously described,^E1^ using metal lancets and commercial allergen extracts (Stallergenes, Antony, France/ALK-Abelló, Hørsholm, Denmark) as well as fresh milk (FM) and raw egg prepared on the day of the SPT. Whole milk was used for SPT to FM. In the SPT to raw egg, the preparation included both egg white and egg yolk. The difference in millimeters between the wheal diameters to FM and milk extract and between raw egg and egg extract was calculated. Specific IgE to milk or egg white was tested using ImmunoCAP (Thermofisher, Uppsala, Sweden). The oral food challenges (OFC) referrals were done at the clinician's discretion, depending on the clinical history and allergy test results.

### Oral food challenges

Patients underwent open OFC using standardized updosing schedules (Table E2). Fresh full fat cow's milk and scrambled egg or omelette cooked on the cooker for 5 minutes were used for FM and cooked egg OFC, respectively. Standardized recipes for milk- and/or egg-containing cupcakes were used for baked milk and baked egg OFCs (Figures E1 and E2). OFCs were considered negative when the child successfully consumed the total cumulative dose without displaying any signs of an allergic reaction and were considered positive if objective signs of an allergic reaction developed. If so, the OFC was stopped, and patients were treated according to local guidelines and at the discretion of the clinician. Tolerant children were recommended to incorporate the tolerated food form into their diet at least twice weekly, and allergic children were recommended to avoid the culprit food.

### Statistical analyses

For determining the significance of differences between groups, the Wilcoxon rank sum test was used. The performance of allergy tests was examined against the allergic status using receiver-operating characteristic curve analyses, with their respective sensitivity, specificity, and predictive positive and negative values. Optimal cutoff points were based on the largest Youden index and the 100% and 50% positive predictive value cutoffs were determined by approximation. Statistical analyses were done using Stata statistical software; release 15 (StataCorp, Collegue Station, Texas), and *P* values of less than .05 were considered statistically significant.
